# Waist circumference and risk of elevated blood pressure in children: a cross-sectional study

**DOI:** 10.1186/1471-2458-11-613

**Published:** 2011-08-02

**Authors:** Cheuk-Sing Choy, Wan-Yu Chan, Ta-Liang Chen, Chun-Chuan Shih, Li-Chu Wu, Chien-Chang Liao

**Affiliations:** 1 Emergency and Intensive Care Department, Taipei Hospital, Department of Health, Executive Yuan, Taiwan; 2 Department of Emergency Medicine, Taipei Medical University Hospital, Taipei 110, Taiwan; 3 Department of Nursing, Min-Hwei College of Health Care Management, Tainan 736, Taiwan; 4 Department of Anesthesiology, Taipei Medical University Hospital, Taipei 110, Taiwan; 5 Department of Anesthesiology, College of Medicine, Taipei Medical University, Taipei 110, Taiwan; 6 The School of Chinese Medicine for Post-Baccalaureate, I-Shou University, Kaohsiung County 82445, Taiwan; 7 Institute of Health Policy and Management, College of Public Health, National Taiwan University, Taipei 100, Taiwan; 8 Management Office for Health Data, China Medical University Hospital, Taichung 404, Taiwan

**Keywords:** Children, obesity, elevated blood pressure, waist circumference

## Abstract

**Background:**

Increasing childhood obesity has become a major health threat. This cross-sectional study reports associations between schoolchildren's waist circumference (WC) and risk of elevated blood pressure.

**Methods:**

We measured height, weight, neck and waist circumference, and blood pressure in regular health examinations among children in grade 1 (ages 6-7 years) at six elementary schools in Taipei County, Taiwan. Elevated blood pressure was defined in children found to have mean systolic or diastolic blood pressure greater than or equal to the gender-, age-, and height-percentile-specific 95th-percentile blood pressure value.

**Results:**

All 2,334 schoolchildren were examined (response rate was 100% in the six schools). The mean of systolic and diastolic blood pressure increased as WC quartiles increased (p < 0.0001). The prevalence of elevated blood pressure for boys and girls within the fourth quartile of waist circumference was 38.9% and 26.8%, respectively. In the multivariate logistic regression analyses, the adjusted odds ratios of elevated blood pressure were 1.78 (95% confidence interval [CI] = 1.13-2.80), 2.45 (95% CI = 1.56-3.85), and 6.03 (95% CI = 3.59-10.1) for children in the second, third, and fourth waist circumference quartiles compared with the first quartile. The odds ratios for per-unit increase and per increase of standard deviation associated with elevated blood pressure were 1.14 (95% CI = 1.10-1.18) and 2.22 (95% CI = 1.76-2.78), respectively.

**Conclusions:**

Elevated blood pressure in children was associated with waist circumference. Not only is waist circumference easier to measure than blood pressure, but it also provides important information on metabolic risk. Further research is needed on effective interventions to identify and monitor children with increased waist circumference to reduce metabolic and blood pressure risks.

## Background

Recent studies have shown significant increases in obesity among Asian and Caucasian children over the past 20 years [1-3]. In Taiwan, the prevalence of obesity among children aged 12-15 was 11.3%, 13.0%, and 13.7% in 1980-1982, 1986-1988, and 1994-1996, respectively [1]. Among children aged 7-13 years in Canada, the prevalence of obesity rose from 5% in 1981 to 15% in 1996 for girls and 17% for boys [2]. In the United States, the prevalence of obesity among children aged 6-11 years was as high as 15.8% in 1999-2002 [3]. Among children and adolescents aged 10-19 years, this is of particular concern because childhood obesity is associated with high risks of hypertension as well as type 2 diabetes, abnormal lipid profiles and early atherosclerosis [4-12]. In addition, childhood obesity is associated with high risk of adult hypertension [13]. Children with elevated blood pressures are at increased risk of hypertension and metabolic syndrome later in life [14].

Pediatric hypertension is increasing along with the pediatric obesity epidemic. Blood pressure measurement has not been included in elementary schoolchildren's health examinations, nor have blood pressure checks been required during pediatric medical visits in Taiwan. This has led to underdiagnosis of pediatric hypertension in clinical settings [15].

Body mass index is a common measure used to identify obesity. However, compared with body mass index, waist circumference (WC) is a better index for investigating metabolic abnormalities such as hypertension and impaired fasting glucose [5-7,16-24]. Maffeis et al. suggested that WC is very helpful in detecting metabolic and cardiovascular risks among overweight children [23]. Although increased WC is well-defined as a risk factor in adults, such studies in children were rare. Few studies have investigated the association between hip circumference and elevated blood pressure [25-27]. Studies investigating the relationship between WC and elevated blood pressure in school-age children also have been lacking. This study thus investigates the association between WC and elevated blood pressure among children aged 6-7 years in Taiwan.

## Methods

### Study design

We conducted health examinations among 7-year-old first-grade children at six public elementary schools in Taipei County, Taiwan. In 2007, 12 of 211 (5.7%) public elementary schools (enrolling 5142 students) in Taipei County cooperated with Taipei Medical University Hospital for regular health examinations. More than 95% of Taiwan's children study at public elementary schools. These 12 public elementary schools were of medium size and located in moderately urbanized areas of mixed socioeconomic status. We randomly chose six of these schools (enrolling 2,447 students) to collected data for this study. The age and sex distribution of children in the six schools was similar to the other six schools. The gender distribution in our study (the proportion of boys was 52.16%) was similar to other studies (52.57%) conducted in Taiwan among children aged 6-7 years [28,29]. Recruitment took place as part of the school's annual health examination, and the study presented no eligibility criteria except willingness to participate. In the past, annual elementary school health examinations included height and weight measurement, an oral check-up, a vision test and some basic medical examinations. This study added neck circumference, waist circumference, and blood pressure measurements to the regular examination after consent was obtained from elementary school administrators and families. All first-grade students at these six elementary schools were examined using the same protocol.

The numbers of first-grade children at schools A, B, C, D, E and F were 329, 642, 300, 368, 634, and 174. Teams consisting of six physicians, two dentists, nine registered nurses and three research assistants conducted these health examinations. About 103 children were excluded from analysis because their values were considered to be incorrect due to data registry error and/or incomplete anthropometric information. There were no significant differences in age or sex between children included (n = 2447) and those not included (n = 2695). This study was approved by the Research Ethics Committee of Taipei Medical University Hospital. Oral informed consent was obtained from parents of examinees.

### Measurements

Children wearing lightweight clothing without shoes were measured for standing height (stadiometer, Bodymeter 208; SECA, Hanover, Germany) and weight (scale, HA-521; Tanita, Tokyo, Japan) [5]. A nonelastic flexible tape measure was used to measure neck, waist, and hip areas without clothing as the subjects stood [5]. In this study, neck circumference was measured midway on the neck, between the mid-cervical spine and mid-anterior neck to within 0.1 cm [30,31]. The smallest circumference between the hip and chest was measured as WC [8]. The hip circumference was measured at the greater trochanter [25-27]. Because boys have larger WC and hip circumference compared with girls [7,10,16,24,25,32], we used sex-specific cutoff points to divide WC and hip circumference into separate quartiles for boys and girls. Waist-to-height ratio was calculated as waist/height [21,23,33]. We used weight (kg) divided by the square of height (m^2^) to calculate body mass index. The instruments (measuring height, weight, and blood pressure) were calibrated according to standard operating prodedures before measuring for anthropometries and blood pressure.

The blood pressure measurement followed the recommendation of the Fourth Report on the Diagnosis, Evaluation and Treatment of High Blood Pressure in Children and Adolescents [34]. In the morning, after students had sat quietly for at least 5 minutes, we measured blood pressure using a mercury sphygmomanometer on the right arm with a cuff that covered more than two-thirds of the upper arm. The first and fifth Korotkoff sounds were recorded as the systolic and diastolic blood pressure. To avoid the effects of white-coat hypertension, blood pressure was measured twice and blood pressure value was taken as the mean of the two measurements. The anthropometry and blood pressure measurements were completed by two trained medical research assistants supervised by a senior medical doctor. There were no significant differences in height, weight, body mass index, WC, hip circumference, and blood pressure values between the two research assistants (operators) except for systolic blood pressure in girls. To avoid the operator effect, we considered the operator as a covariate in the final model. Elevated blood pressure was defined in students found to have either mean systolic or diastolic blood pressure greater than or equal to the gender-, age-, and height-percentile-specific 95th-percentile blood pressure value according to the criteria of the National High Blood Pressure Education Program Working Group on Hypertension Control in Children and Adolescents [35].

### Statistical analysis

Kruskal-Wallis tests were used to compare the means of height, weight, body mass index, neck circumference, WC, hip circumference, and blood pressure between quartiles of WC by sex. We used Chi-square tests to compare the proportion of elevated systolic blood pressure, elevated diastolic blood pressure, and elevated blood pressure between quartiles of WC circumference. Pearson's correlation coefficients were used to investigate the correlations between WC and height, neck circumference, hip circumference, body mass index, and blood pressure. Sex-specific odds ratios (ORs) and corresponding 95% confidence intervals (CIs) for elevated blood pressure in association with WC and hip circumference were calculated in multivariate logistic regression analysis adjusted for age, sex, operator, height, and body mass index. For further analysis, we used per-unit increase and per-standard deviation (SD) increase in hip circumference, waist-to-height ratio and body mass index to predict elevated blood pressure in the multivariate logistic regression analysis, and calculated corresponding ORs and 95% CIs. We also estimated the sex-specific sensitivity specificity, positive predictive value and negative predictive value of WC for predicting elevated blood pressure. All analyses were performed with SAS software, version 8.0 (SAS Institute Inc., Carey, NC, USA). Two-sided probability value < 0.05 was considered statistically significant.

## Results

All anthropometric measurements, including height, weight, body mass index, neck circumference and hip circumference, and all measures of blood pressure, including systolic blood pressure, diastolic blood pressure, and elevated blood pressure, increased with increasing waist circumference (Table 1).

**Table 1:** Characteristics of children aged 6-7 years by quartiles of waist circumference

	Quartiles				
		
	1^st^	2^nd^	3^rd^	4^th^	
		
	Mean ± SD	Mean ± SD	Mean ± SD	Mean ± SD	*p*-value
Boys					
Number	381	286	257	288	
Age, years	6.4 ± 0.3	6.4 ± 0.3	6.5 ± 0.5	6.4 ± 1.4	0.0007
Height, cm	117.6 ± 4.6	119.3 ± 4.4	121.2 ± 4.8	123.4 ± 4.5	< 0.0001
Weight, kg	20.9 ± 2.4	22.5 ± 2.4	24.8 ± 3.1	31.0 ± 5.3	< 0.0001
BMI, kg/m^2^	15.1 ± 1.2	15.8 ± 1.3	16.9 ± 1.7	20.3 ± 2.9	< 0.0001
NC, cm	25.6 ± 1.1	26.3 ± 1.8	27.2 ± 1.3	29.2 ± 1.9	< 0.0001
WC, cm	52.2 ± 1.7	56.0 ± 0.8	59.4 ± 1.1	67.8 ± 5.8	< 0.0001
HC, cm	63.4 ± 3.1	66.1 ± 2.8	69.1 ± 5.1	76.7 ± 5.4	< 0.0001
SBP, mmHg	95.0 ± 13.8	97.1 ± 13.3	103.6 ± 13.2	110.3 ± 15.6	< 0.0001
DBP, mmHg	58.0 ± 11.4	60.1 ± 11.8	64.2 ± 11.6	68.4 ± 13.6	< 0.0001
E-SBP, %	7.6	10.1	17.5	30.9	< 0.0001
E-DBP, %	7.1	8.7	14.4	21.9	< 0.0001
EBP, %	11.0	15.0	24.5	38.9	< 0.0001
Girls					
Number	294	286	273	269	
Age, years	6.4 ± 0.29	6.5 ± 0.5	6.5 ± 0.3	6.5 ± 0.4	0.0747
Height, cm	116.3 ± 4.78	117.9 ± 4.6	119.3 ± 4.5	122.1 ± 4.8	< 0.0001
Weight, kg	19.9 ± 2.59	21.4 ± 2.3	23.2 ± 2.8	28.1 ± 4.8	< 0.0001
BMI, kg/m^2^	14.7 ± 1.23	15.4 ± 1.3	16.3 ± 1.5	18.8 ± 2.5	< 0.0001
NC, cm	24.8 ± 1.04	25.3 ± 1.1	25.9 ± 1.8	27.5 ± 1.6	< 0.0001
WC, cm	50.3 ± 1.75	54.1 ± 0.8	57.3 ± 1.1	64.1 ± 4.2	< 0.0001
HC, cm	62.8 ± 3.08	65.2 ± 4.6	68.2 ± 3.3	73.9 ± 5.0	< 0.0001
SBP, mmHg	92.2 ± 14.6	95.2 ± 13.3	98.2 ± 14.0	103.0 ± 13.5	< 0.0001
DBP, mmHg	57.2 ± 10.8	58.4 ± 11.0	61.3 ± 12.6	64.0 ± 12.2	< 0.0001
E-SBP, %	5.1	8.0	10.3	19.7	< 0.0001
E-DBP, %	5.1	6.6	8.8	15.2	< 0.0001
EBP, %	6.8	11.9	13.6	26.8	< 0.0001

BMI, body mass index; NC, neck circumference; WC, waist circumference; HC, hip circumference; SBP, systolic blood pressure; DBP, diastolic blood pressure; E-SBP, elevated systolic blood pressure; E-DBP, elevated diastolic blood pressure; EBP, elevated blood pressure

**Figure 1:**
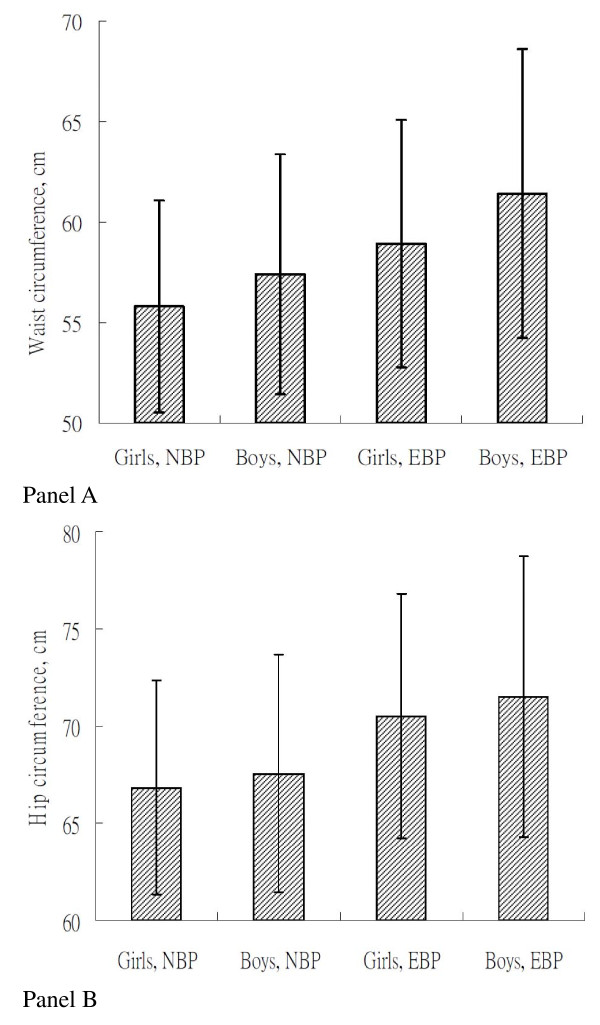
**Means of waist circumference and hip circumference by sex and status of elevated blood pressure**. (NBP, normal blood pressure; EBP, elevated blood pressure); *P *< 0.0001 among four groups in both panels.

As shown in Figure 1, within each sex, children with high BP had higher WC than those with normal BP. The mean of hip circumference was higher in children with elevated blood pressure than in children with normal blood pressure both in boys and girls. Table 2 shows that WC is positively correlated with height, body mass index, hip circumference, and blood pressure in both girls and boys. The high correlations between hip circumference and blood pressure were also noted in boys and girls. Height is also positively correlated with systolic and diastolic blood pressure in boys and girls.

**Table 2:** Pearson's correlation coefficient between anthropometrics and blood pressure by sex

	Height, cm	NC, cm	WC, cm	HC, cm	BMI, kg/m^2^	SBP, mmHg	DBP, mmHg
Boys							
Height, cm	1						
NC, cm	0.429	1					
WC, cm	0.456	0.726	1				
HC, cm	0.541	0.706	0.844	1			
BMI, kg/m^2^	0.337	0.728	0.814	0.807	1		
SBP, mmHg	0.178	0.305	0.377	0.375	0.321	1	
DBP, mmHg	0.155	0.238	0.316	0.300	0.233	0.683	1
Girls							
Height, cm	1						
NC, cm	0.450	1					
WC, cm	0.456	0.656	1				
HC, cm	0.556	0.647	0.792	1			
BMI, kg/m^2^	0.347	0.646	0.763	0.748	1		
SBP, mmHg	0.194	0.252	0.295	0.340	0.211	1	
DBP, mmHg	0.135	0.169	0.224	0.229	0.132	0.678	1

All at significant difference (p < 0.0001)

BMI, body mass index; HC, hip circumference; NC, neck circumference; WC, waist circumference; SBP, systolic blood pressure; DBP, diastolic blood pressure

In the multivariate logistic regression, elevated blood pressure was associated with quartiles, per-unit increase, and per-SD increase of WC both in girls and boys (Table 3). The corresponding ORs of elevated blood pressure associated with quartiles, per-unit increase, and per-SD increase of hip circumference were also noted in girls and boys. Per-unit and per-SD increase in waist-to-height ratio were associated with risk of elevated blood pressure. However, there was no significant association between body mass index (per-unit increase or per-SD increase) and elevated blood pressure after adjusting covariates.

**Table 3:** Adjusted odds ratio and 95% confidence intervals of elevated blood pressure associated with anthropometrics among schoolchildren

	Model 1 OR (95% CI)	Model 2 OR (95% CI)	Model 3 OR (95% CI)
Quartiles of WC*			
1^st^	1.00 (Reference)	1.00 (Reference)	1.00 (Reference)
2^nd^	1.73 (1.11-2.71)	1.77 (1.13-2.77)	1.78 (1.13-2.80)
3^rd^	2.33 (1.51-3.58)	2.41 (1.55-3.76)	2.45 (1.56-3.85)
4^th^	5.42 (3.66-8.04)	5.79 (3.76-8.91)	6.03 (3.59-10.1)
*p *for trend	< 0.0001	< 0.0001	< 0.0001
Quartiles of HC*			
1^st^	1.00 (Reference)	1.00 (Reference)	1.00 (Reference)
2^nd^	1.29 (0.84-2.00)	1.38 (0.88-2.16)	1.38 (0.88-2.16)
3^rd^	2.27 (1.51-3.40)	2.57 (1.66-3.99)	2.55 (1.61-4.06)
4^th^	4.40 (2.97-6.50)	5.28 (3.32-8.42)	5.18 (2.87-9.36)
*p *for trend	< 0.0001	< 0.0001	< 0.0001
WC per unit increase	1.10 (1.08-1.12)	1.10 (1.08-1.13)	1.14 (1.10-1.18)
WC per SD increase	1.77 (1.56-2.00)	1.82 (1.58-2.10)	2.22 (1.76-2.78)
HC per unit increase	1.10 (1.08-1.12)	1.12 (1.09-1.15)	1.20 (1.14-1.26)
HC per SD increase	1.81 (1.59-2.07)	2.00 (1.70-2.36)	3.03 (2.23-4.11)
WHtR per 0.01 increase	1.13 (1.10-1.16)	1.13 (1.10-1.16)	1.17 (1.12-1.23)
WHtR per SD increase	1.74 (1.54-1.98)	1.71 (1.51-1.94)	2.03 (1.66-2.48)

HC, hip circumference; WC, waist circumference; OR, odds ratio; CI, confidence interval; SD, standard deviation; WHtR, waist-to-height ratio

Model 1: adjusted for age, sex, and operator

Model 2: adjusted for age, sex, operator, and height

Model 3: adjusted for age, sex, operator, height, and body mass index

The best cutoff points for WC to predict elevated blood pressure for boys and girls were 59 cm (sensitivity = 62.69%, specificity = 67.75%, positive predictive value = 34.5%, negative predictive value = 86.9%) and 57 cm (sensitivity = 62.58%, specificity = 63.61%, positive predictive value = 22.6%, negative predictive value = 90.9%), respectively (not shown in the tables).

## Discussion

This study meticulously investigated the relationship between WC and elevated blood pressure among children aged 6-7 years after adjustment for age, sex, operator, height, and body mass index. Children with elevated blood pressure had higher mean WC than those with normal blood pressure. Over a quarter of the children (26.8%) in the highest WC quartile had elevated blood pressure, revealing that high WC is a risk factor for elevated blood pressure in children aged 6-7 years.

Associations between elevated blood pressure and WC have been documented among children in Mexico, Greece and the United States [21,36,37]. Stratification of WC into quartiles may be useful in investigating elevated blood pressure. In our study, higher risk for elevated blood pressure was also found in children in the highest quartile than in children within the lower quartiles. A previous study found that with one cm incremental increase in WC, the OR of elevated blood pressure was 1.06 (95% CI = 1.01-1.11) [10]. However, we demonstrate a more significant OR in our study (OR = 1.14, 95% CI = 1.10-1.18).

In contrast with another study [38], we found elevated blood pressure had a higher association with WC than body mass index. Previous studies considered body mass index as a covariate in the association between waist circumference and hypertension [9,18]. Among prepubertal children in the present study, waist circumference *per se *may be a useful parameter to predict elevated blood pressure independently of body mass index. Although body mass index and WC have a very high correlation coefficient and may be used interchangeably, our study found a significant association between large WC and elevated BP after adjusting for body mass index. Body mass index represented whole-body obese status, while WC was considered an indicator of central obesity or abdominal obesity. Compared with body mass index it is likely that WC is more important, because it has a greater association with metabolic syndrome and cardiovascular diseases [39].

The relation between childhood cardiovascular risk factors and metabolic syndrome is undeniable [40]. Among children and adolescents in US, mean WC and prevalence of abdominal obesity both greatly increased between 1988-1994 and 1999-2004 [16]. Increased WC may play a crucial role in cardiovascular disease and metabolic syndrome. A cut-off point of 1.3 standard deviation score of WC was suggested by Fredriks et al. to screen for increased abdominal fat mass in Dutch children [32]. Hirschler et al. showed that WC was a predictor of insulin resistance in children and could be included in clinical practice as a simple tool to help identify children at risk [5]. Combining body mass index and WC could increase the prediction of elevated blood pressure and metabolic syndrome; it could also be used in clinical settings to evaluate risks to children's health [36]. In Italy, obese children with WC > 90th percentile had a higher risk for metabolic syndrome (OR = 13.1) [23]. This shows waist circumference measurement may help identify children at risk for metabolic syndrome [41]. Waist circumference also seems to be the best predictor of metabolic syndrome in pediatric clinical settings [6].

Simple anthropometric measurements are the most commonly used and practical tools for assessing body composition [22]. WC is a simple measure of abdominal obesity and predicts total fat content well in children [16,22]. Compared with body mass index, abdominal obesity may be a better predictor than overall obesity of hypertension and metabolic abnormalities [5,7,16-21,24]. WC is much easier to measure than blood pressure in terms of training and access to equipment, especially in low-income settings. Blood pressure measurement requires greater operator skill, and blood pressure is liable to be falsely elevated unless measured with care and in stress-free situations. Because WC is significantly correlated with blood pressure, we suggest measurement of WC as a screening tool for elevated blood pressure in children. Our study's moderate sensitivity and specificity suggest that using WC to screen for elevated blood pressure in children should be performed carefully. Some children with elevated blood pressure may not have large WC, and vice versa.

In addition, waist-to-height ratio is another index to measure abdominal obesity. Much evidence suggests waist-to-height ratio is associated with elevated blood pressure [21,23,33]. In our study, the association between WC and elevated blood pressure is greater than the association between waist-to-height ratio and elevated blood pressure after adjustment for age, sex, operator, height, and body mass index.

Compared to WC, the association between hip circumference and elevated blood pressure was poorly understood. Previous studies associated larger hip circumference with reduced risk of elevated blood pressure in adults [25-27], but studies investigating the association in children are lacking. Our study suggests an independent association between pediatric hip circumference and elevated blood pressure, and that hip circumference is as good as WC in predicting elevated blood pressure in children.

### Limitations

We have no information on family history of cardiovascular diseases, intake of sugar-added beverages, or nutritional status and lifestyle in these elementary school children. These factors are associated with childhood hypertension [42-44]. Another limitation of this study is that we excluded 103 children from the analysis because of incorrect data entry or anthropometric information. Furthermore, our cross-sectional study could not infer causation. Despite this study's limitations, our study could be generalized to the same-age population in Taiwan, and the significant findings suggest that increased WC may predict elevated blood pressure in children.

## Conclusions

This study showed that elevated blood pressure in children was associated with waist circumference, which is both easier to measure than blood pressure and provides important information on metabolic risk. We also found that hip circumference is as good as WC in predicting elevated blood pressure in children. This suggests the need to monitor elevated blood pressure in childhood by taking regular WC and blood pressure measurements during school health examinations. Further research is needed into the effectiveness of interventions to monitor waist circumference to reduce BP and metabolic risks in children.

## Abbreviations

**CI**: confidence interval; **OR**: odds ratio; **SD**: standard deviation; **WC**: waist circumference

## Competing interests

The authors declare that they have no competing interests.

## Authors' contributions

CSC participated in data management and interpretation, drafting the manuscript, and substantial revision of the article. WYC participated in study design, data analysis, data management and interpretation, and substantial revisions. TLC participated in the literature review, drafting the manuscript, data management, interpretation of the data, and substantial revisions. CCS participated in study design, data analysis, data management, interpretation of the data, and substantial revisions. WLC participated in study design, data analysis, data management, interpretation of the data, and substantial revisions. CCL participated in literature review, study design, data management, data analysis, interpretation of the data, drafting the manuscript and substantial revisions. All authors read and approved the final manuscript.

## Pre-publication history

The pre-publication history for this paper can be accessed here:

http://www.biomedcentral.com/1471-2458/11/613/prepub

## References

[B1] Chu NF Prevalence and trends of obesity among schoolchildren in Taiwan: the Taipei Children's Heart Study Int J Obes 2001 25 170 176 10.1038/sj.ijo.0801486 11410816

[B2] Spurgeon D Childhood obesity in Canada Br Med J 2001 324 1416 10.1136/bmj.324.7351.1416/f PMC1172197 12068865

[B3] Hedley AA Ogden CL Johnson CL Carroll MD Curtin LR Flegal KM Prevalence of overweight and obesity among US children, adolescents and adults, 1999-2002 JAMA 2004 291 2847 2850 10.1001/jama.291.23.2847 15199035

[B4] Sorof JM Lai D Turner J Poffenbarger T Portman RJ Overweight, ethnicity, and the prevalence of hypertension in school-aged children Pediatrics 2004 113 475 482 10.1542/peds.113.3.475 14993537

[B5] Hirschler V Aranda C Calcagno ML Maccalini G Jadzinsky M Can waist circumference identify children with the metabolic syndrome? Arch Pediatr Adolesc Med 2005 159 740 744 10.1001/archpedi.159.8.740 16061781

[B6] Moreno LA Pineda I Rodriguez G Fleta J Sarría A Bueno M Waist circumference for the screening of metabolic syndrome in children Acta Paediatr 2002 91 1307 1312 10.1111/j.1651-2227.2002.tb02825.x 12578286

[B7] Genovesi S Antolini L Giussani M Pieruzzi F Galbiati S Valsecchi MG Brambilla P Stella A Usefulness of waist circumference for the identification of childhood hypertension J Hypertens 2008 26 1563 1570 10.1097/HJH.0b013e328302842b 18622233

[B8] Reich A Müller G Gelbrich G Deutscher K Gödicke R Kiess W Obesity and blood pressure: results from the examination of 2365 schoolchildren in Germany Int J Obes 2003 27 1459 1464 10.1038/sj.ijo.0802462 14634675

[B9] Grievink L Alberts JF O'Niel J Gerstenbluth I Waist circumference as a measurement of obesity in the Netherlands Antilles: associations with hypertension and diabetes mellitus Eur J Clin Nutr 2004 58 1159 1165 10.1038/sj.ejcn.1601944 15054429

[B10] Colín-Ramírez E Castillo-Martínez L Orea-Tejeda A Villa Romero AR Vergara Castañeda A Asensio Lafuente E Waist circumference and fat intake are associated with high blood pressure in Mexican children aged 8 to 10 years J Am Diet Assoc 2009 109 996 1003 10.1016/j.jada.2009.03.011 19465181

[B11] Liao CC Su TC Chien KL Wang JK Chiang CC Lin CC Lin RS Lee YT Sung FC Elevated blood pressure, obesity, and hyperlipidemia J Pediatr 2009 155 79 83 10.1016/j.jpeds.2009.01.036 19446850

[B12] Freedman DS Patel DA Srinivasan SR Chen W Tang R Bond MG Berenson GS The contribution of childhood obesity to adult carotid intima-media thickness: the Bogalusa Heart Study Int J Obes 2008 32 749 756 10.1038/sj.ijo.0803798 18227845

[B13] Li L Law C Power C Body mass index throughout the life-course and blood pressure in mid-adult life: a birth cohort study J Hypertens 2007 25 1215 1223 10.1097/HJH.0b013e3280f3c01a 17563534

[B14] Sun SS Grave GD Siervogel RM Pickoff AA Arslanian SS Daniels SR Systolic blood pressure in childhood predicts hypertension and metabolic syndrome later in life Pediatrics 2007 119 237 246 10.1542/peds.2006-2543 17272612

[B15] Hansen ML Gunn PW Kaelber DC Underdiagnosis of hypertension in children and adolescents JAMA 2007 298 874 879 10.1001/jama.298.8.874 17712071

[B16] Li C Ford ES Mokdad AH Cook S Recent trends in waist circumference and waist-height ratio among US children and adolescents Pediatrics 2006 118 e1390 e1398 10.1542/peds.2006-1062 17079540

[B17] Carotenuto M Bruni O Santoro N Del Giudice EM Perrone L Pascotto A Waist circumference predicts the occurrence of sleep-disordered breathing in obese children and adolescents: a questionnaire-based study Sleep Med 2006 7 357 361 10.1016/j.sleep.2006.01.005 16713341

[B18] Maffeis C Pietrobelli A Grezzani A Provera S Tatò L Waist circumference and cardiovascular risk factors in prepubertal children Obes Res 2001 9 179 187 10.1038/oby.2001.19 11323443

[B19] Barba G Troiano E Russo P Strazzullo P Siani A Body mass, fat distribution and blood pressure in Southern Italian children: results of the ARCA project Nutr Metab Cardiovasc Dis 2006 16 239 248 10.1016/j.numecd.2006.02.005 16679215

[B20] Maffeis C Grezzani A Pietrobelli A Provera S Tatò L Does waist circumference predict fat gain in children? Int J Obes 2001 25 978 983 10.1038/sj.ijo.0801641 11443495

[B21] Savva SC Tornaritis M Savva ME Kourides Y Panagi A Silikiotou N Georgiou C Kafatos A Waist circumference and waist-to-height ratio are better predictors of cardiovascular disease risk factors in children than body mass index Int J Obes 2000 24 1453 1458 10.1038/sj.ijo.0801401 11126342

[B22] Sarria A Moreno LA Garcia-Llop LA Fleta J Morellón MP Bueno M Body mass index, triceps skinfold and waist circumference in screening for adiposity in male children and adolescents Acta Paediatr 2001 90 387 392 11332928 10.1080/080352501750126195

[B23] Maffeis CM Banzato C Talamini G Waist-height ratio, a useful index to identify high metabolic risk in overweight children J Pediatr 2008 152 207 213 10.1016/j.jpeds.2007.09.021 18206690

[B24] Katzmarzyk PT Scrinivasan SR Chen W Malina RM Bouchard C Berenson GS Body mass index, waist circumference, and clustering of cardiovascular disease risk factors in a biracial sample of children and adolescents Pediatrics 2004 114 e198 e205 10.1542/peds.114.2.e198 15286257

[B25] Snijder MB Zimmet PZ Visser M Dekker JM Seidell JC Shaw JE Independent and opposite associations of waist and hip circumferences with diabetes, hypertension and dyslipidemia: the AusDiab Study Int J Obes 2004 28 402 409 10.1038/sj.ijo.0802567 14724659

[B26] Esmaillzadeh A Mirmiran P Moeini SH Azizi F Larger hip circumference independently contributed to reduced metabolic risks in Tehranian adult women Int J Cardiol 2006 108 338 345 10.1016/j.ijcard.2005.05.019 15963581

[B27] Esmaillzadeh A Mirmiran P Azadbakht L Amiri P Azizi F Independent and inverse association of hip circumference with metabolic risk factors in Tehranian adult men Prev Med 2006 42 354 357 10.1016/j.ypmed.2005.12.009 16545445

[B28] Ng KC Lai SW Application of anthropometric indices in childhood obesity South Med J 2004 97 566 570 10.1097/00007611-200406000-00011 15255423

[B29] Chuang SY Pan WH Predictability and implications of anthropometric indices for metabolic abnormalities in children: nutrition and health survey in Taiwan elementary children, 2001-2002 Asia Pac J Clin Nutr 2009 18 272 279 19713188

[B30] Ben-Noun LL Laor A Relationship between changes in neck circumference and changes in blood pressure Am J Hypertens 2004 17 409 414 10.1016/j.amjhyper.2004.02.005 15110899

[B31] Onat A Hergenç G Yüksel H Can G Ayhan E Kaya Z Dursunoğlu D Neck circumference as a measure of central obesity: associations with metabolic syndrome and obstructive sleep apnea syndrome beyond waist circumference Clin Nutr 2009 28 46 51 10.1016/j.clnu.2008.10.006 19010573

[B32] Fredriks AM van Buuren S Fekkes M Verloove-Vanhorick SP Wit JM Are age references for waist circumference, hip circumference and waist-hip ratio in Dutch children useful in clinical practice? Eur J Pediatr 2005 164 216 222 10.1007/s00431-004-1586-7 15662504

[B33] Hara M Saitou E Iwata F Okada T Harada K Waist-to-height ratio is the best predictor of cardiovascular disease risk factors in Japanese schoolchildren J Atheroscler Thromb 2002 9 127 132 10.5551/jat.9.127 12226553

[B34] National High Blood Pressure Education Program Working Group on High Blood Pressure in Children and Adolescents The Fourth Report on the Diagnosis, Evaluation, and Treatment of High Blood Pressure in Children and Adolescents Pediatrics 2004 114 555 576 15286277

[B35] National High Blood Pressure Education Program Working Group on Hypertension Control in Children and Adolescents Update on the 1987 Task Force Report on High Blood Pressure in Children and Adolescents: a working group report from the National High Blood Pressure Education Program Pediatrics 1996 98 649 658 8885941

[B36] Janssen I Katzmarzyk PT Srinivasan SR Chen W Malina RM Bouchard C Berenson GS Combined influence of body mass index and waist circumference on coronary artery disease risk factors among children and adolescents Pediatrics 2005 115 1623 1616 10.1542/peds.2004-2588 15930225

[B37] Perichart-Perera O Balas-Nakash M Schiffman-Selechnik E Barbato-Dosal A Vadillo-Ortega F Obesity increases metabolic syndrome risk factors in school-aged children from an urban school in Mexico City J Am Diet Assoc 2007 107 81 91 10.1016/j.jada.2006.10.011 17197275

[B38] Morimoto A Nishimura R Kanda A Sano H Matsudaira T Miyashita Y Shirasawa T Takahashi E Kawaguchi T Tajima N Waist circumference estimation from BMI in Japanese children Diabetes Res Clin Pract 2007 75 96 98 10.1016/j.diabres.2006.05.022 16945447

[B39] Hirschler V Maccallini G Calcagno M Aranda C Jadzinsky M Waist circumference identifies primary school children with metabolic syndrome abnormalities Diabetes Technol Ther 2007 9 149 157 10.1089/dia.2006.0017 17425440

[B40] Reis EC Kip KE Marroquin OC Kiesau M Hipps L Jr Peters RE Reis SE Screening children to identify families at increased risk for cardiovascular disease Pediatrics 2006 118 e1789 e1797 10.1542/peds.2006-0680 17142500

[B41] Sellers EAC Singh GR Sayers SM Large waist but low body mass index: the metabolic syndrome in Australian aboriginal children J Pediatr 2008 153 222 227 10.1016/j.jpeds.2008.02.006 18534223

[B42] Giampietro O Virgone E Carneglia L Griesi E Calvi D Matteucci E Anthropometric indices of schoolchildren and familiar risk factors Prev Med 2002 35 492 498 10.1006/pmed.2002.1098 12431897

[B43] Linardakis M Sarri K Pateraki MS Sbokos M Kafatos A Sugar-added beverages consumption among kindergarten children of Crete: effects on nutritional status and risk of obesity BMC Public Health 2008 8 279 10.1186/1471-2458-8-279 18684334 PMC2525654

[B44] Longo-Mbenza B Luila EL M'Buyamba-Kabangu JR Nutritional status, socio-economic status, heart rate, and blood pressure in African schoolchildren and adolescents Int J Cardiol 2007 121 171 177 10.1016/j.ijcard.2006.11.004 17258822

